# ‘Like walking with someone as opposed to trying to catch up to them’—Dynamics at play when clinicians and young people formulate together

**DOI:** 10.1111/papt.12543

**Published:** 2024-09-06

**Authors:** Laura J. Douglas, Cian Aherne, Patrick Ryan, Barry Coughlan, Donal G. Fortune

**Affiliations:** ^1^ Psychology Department University of Limerick Limerick Ireland; ^2^ Jigsaw Youth Mental Health Services Limerick Ireland

**Keywords:** case conceptualisation, formulation, psychotherapy, therapeutic processes, transitional aged youth

## Abstract

**Objective:**

The aim of the present study was to explore the social process of formulation in talk therapy between young people and clinicians.

**Design:**

Qualitative semi‐structured interview study.

**Method:**

Ten young people (male = 6, female = 4, age range = 16–23 years) and nine clinicians from various disciplines within a youth mental health service were interviewed. Constructivist grounded theory was used for the analysis.

**Results:**

Four themes were constructed from the data; a ‘level playing field’ between young person and clinician enables formulation, formulating is a constant process of getting it right and getting it wrong, emotional expression and attunement get us closer to each other and to understanding, and ‘formulation versus diagnosis’ can create tension in the therapy room. The constructivist grounded theory devised demonstrated how the dynamics of power, collaboration, openness, and the therapeutic relationship are constantly in flux during the process of formulation.

**Conclusion:**

The paper presents a constructivist grounded theory which incorporates dynamics relating to power, collaboration, and openness. The importance of the therapeutic relationship is also emphasised. The theory encourages continuous and recursive personal reflection by the therapist as to how they can be optimally attuned to the dynamics of power, collaboration, and openness with young people.

**Practitioner points**

- Dynamics of power, collaboration, and openness inform the social process of formulation.
- The therapeutic relationship acts as a ‘tuning fork’, honing in on the needs of the young person.
- Model proposed has scope for use as reflective practice tool in supervision spaces.

## INTRODUCTION

Formulation is generally considered to be a joint enterprise between a client and a therapist to develop a shared understanding and plan for the work to be conducted. At its best, the process generates an atmosphere of ‘reciprocal learning’ for both the client and therapist (Ryan, 2019, p. 6). Formulation is widely used and has been shown to reduce distress and improve the therapeutic alliance (Nattrass et al., 2015); however, it is an under‐researched phenomenon, despite its widespread application in mental health services (Johnstone & Dallos, 2014). Clients report largely positive perspectives on the process of formulation, although the experience can be challenging (Chadwick et al., 2003; Evans & Parry, 1996; Gibbs et al., 2020; Halpin et al., 2016; Kahlon et al., 2014; Lewis‐Morton et al., 2017; Pain et al., 2008; Rayner et al., 2011; Redhead et al., 2015; Shine & Westacott, 2010; Spencer et al., 2022; Thew & Krohnert, 2015; Tyrer & Masterson, 2019). Formulation can be considered an ongoing process, rather than a one‐off event or end‐product (Challoner & Papayianni, 2018), and while the inter‐rater reliability of formulations has been well researched (Flinn et al., 2015), there is a clear need to gain the perspectives of stakeholders on how clients and therapists view the process of formulation (Aston, 2009; Easden & Kazantzis, 2018; Thrower et al., 2024).

Four other studies have qualitatively explored both clients’ and clinicians’ perspectives on formulation (Chadwick et al., 2003; Gibbs et al., 2020; Lewis‐Morton et al., 2017; Pain et al., 2008). It is indicated that more work is needed to understand the key interactions and transactions between client and clinician as they formulate together in the therapy room. Formulation relating to young people specifically is under‐theorised, and it is likely that young people and experienced clinicians may speak about the construct of formulation differently. To the author's knowledge, Halpin et al.'s (2016) paper is the only study thus far which explores young people's experiences of formulation, more specifically three young people who had experienced psychosis and were treated using cognitive behavioural therapy (CBT). The resultant interpretative phenomenological analysis (IPA) posits formulation as a challenging process wherein a young person develops insight, broadens their awareness of ‘triggers’, and makes links between past and present. This paper also presents the young people's clinicians’ thoughts around formulation which ranged from how the therapist facilitates finding patterns, to how an intervention might solely be comprised of a formulation as the process of formulation is therapeutic in and of itself. From a methodological perspective, this paper treated the young person and clinician data separately and so there is scope to theorise how both parties' experiences of formulation interact with each other, as formulation is an inherently social process.

The present paper was concerned with how *transitional aged youth* and clinicians experience the inherently social process of formulation. Transitional aged youth refers to a period of emerging adolescence from 15 to 25 years (Wilens & Rosenbaum, 2013). Transitional aged youth are a noteworthy cohort in that they are on the cusp of adulthood, but not quite adults. As noted by Khetarpal et al. (2022), they are gaining independence, forming their identities, and transitioning from second‐level to third level education. They are also transitioning from children's services to adult services, and with this comes increased responsibility. No current empirically developed theory exists relating to this particular cohort's experience of formulation, consequently there was a gap in the literature in this regard. There has been one previous grounded theory study relating to the process of formulation exclusively for adults presenting with an array of self‐reported difficulties (Rayner et al., 2011) which presented a grounded theory model of ‘Doing with’ based on themes of ‘being with the therapist’, ‘understanding and feeling’, and ‘keeping it real’, along with the value of cognitive analytic therapy (CAT) tools. Following this, Gibbs et al. (2020) proposed a grounded theory of how service‐users' experience and make use of formulations relating to psychosis, wherein it was put forth that formulation is built on two core processes: ‘linking previous experiences with current ways of being’ and ‘building the therapeutic relationship’. If these processes are *in situ*, the client can then go on to ‘make use of new understandings’. As transitional aged youth occupy the liminal space between childhood and adulthood, it was hypothesised that formulating with this population may touch upon unique dynamics not previously acknowledged in the literature.

No one mental health presentation nor therapeutic orientation was studied, rather the process of formulation across various presentations and schools of therapy was the focus of this research. Challenges have presented in defining formulation, with a recent consensus study (Thrower et al., 2024) reporting disparity in how clinicians conceptualise formulation. For the purpose of the present study, formulation is defined as ‘a recursive process of suggestion, discussion, reflection, feedback, and revision that is part of the moment‐to‐moment process of therapy’ (Johnstone & Dallos, 2014, p. 4). In this way, formulation will be considered a process, as opposed to a product/event. Social constructivism is the epistemological stance of the paper, a position which has been said to be ‘notoriously slippery and difficult to pin down precisely’ (Hay, 2016, p. 520) a sentiment which mirrors the seemingly indefinable nature of formulation. The present study aims to answer the research question: What dynamics underpin the social process of formulation between young people and clinicians?

## METHOD

### Design

A qualitative methodology was used. The researcher was particularly interested in how clinicians facilitated formulation, how this was received by young people, and how the relationship impacted formulation for both parties. Constructivist grounded theory (CGT, Charmaz, 2014, 2017, 2020) was applied for the analysis.

### Ethics

Ethical approval was granted by the research ethics committee of the national youth mental health service from which participants were recruited. Participation in the research was voluntary and informed written consent was provided by all participants (clinicians, young people, and a parent/guardian if the young person was under 18 years).

### Setting

The research was undertaken in a national youth mental health service. This service offers mental health support to young people aged 12–25 years via a brief intervention model. It is a transdisciplinary service, employing psychologists, mental health nurses, social workers, psychotherapists, and occupational therapists (OTs). It was understood that some young people who participated in the study may not have been explicitly told in therapy that they had experienced ‘the process of formulation’, but that they had undoubtedly engaged with 6–8 sessions of therapy that had a focus on meaning‐making and understanding in relation to the self and personal contexts.

### Inclusion/exclusion criteria

The following were the inclusion/exclusion criteria for participants:

For inclusion in the study, young people had to be aged between 15 and 25 years with mild to moderate mental health difficulties, and had to have completed their period of intervention. Attendance at a brief intervention of formulation‐centred talk therapy which had a formalised ending was indicative of treatment completion. Young people were excluded from the study if a treating clinician deemed their distress to be incompatible with participation in the study.

Clinicians were eligible for inclusion in the study where they had been working in a client facing clinical role for a minimum of 6+ months. Clinicians in their probation phase of employment were not eligible.

### Recruitment

Purposive sampling was used to recruit young people and clinicians with the above characteristics relating to age and experiences. The information pack was emailed to clinical managers in the host organisation's network and they were asked to circulate to their respective teams. It was not a prerequisite that clinician/young person dyads had to participate, ie: young people and clinicians were interviewed regardless of the participation status of their clinician/young person.

#### Recruitment of clinicians

Clinicians were invited to email, phone, or text the primary researcher if they were interested in participating. If a clinician was happy to participate, a Microsoft Teams video call was scheduled. Participation was confidential. Prior to participation, clinicians provided written informed consent.

#### Recruitment of young people

Clinicians were invited to introduce the research to young people following the completion of a brief intervention, and to give young people the research information pack. If a young person was interested in participating, they were asked for verbal consent for their contact details (email or phone) to be passed on to the primary researcher. If a young person was under 18 years, clinicians sought consent from the young person's parent/guardian to relay contact details to the primary researcher. The primary researcher then got in contact with the young person or parent/guardian and described the research project and what participation involved. The young person or parent/guardian was given the opportunity to ask questions about the research. Participants then emailed their signed consent form to the primary researcher and a Microsoft Teams video call was scheduled. Recruitment commenced in July 2021 and ceased in December 2021.

### Participation and procedures

Participation involved a one‐off semi‐structured interview. Being the most recent CGT study in the area, Gibbs et al.'s (2020) topic guide was used in the development of the topic guide. It was reasoned that the topic guides would look different for young people and clinicians, as young people would be reflecting on their own experience specifically, whereas clinicians would be reflecting on their experience of facilitating formulation. Free flowing conversation was encouraged, and it was preferential that these topic areas were addressed naturally throughout the interview, rather than adhering strictly to the order of the protocol.

Interview duration ranged from 21 to 60+ minutes, were facilitated via Microsoft Teams, and were audio recorded. The primary researcher transcribed and anonymised the data. Participants were given the option for their contact details to be held by the primary researcher to forward on the finalised paper arising from the interviews.

### Analysis

**FIGURE 1:**
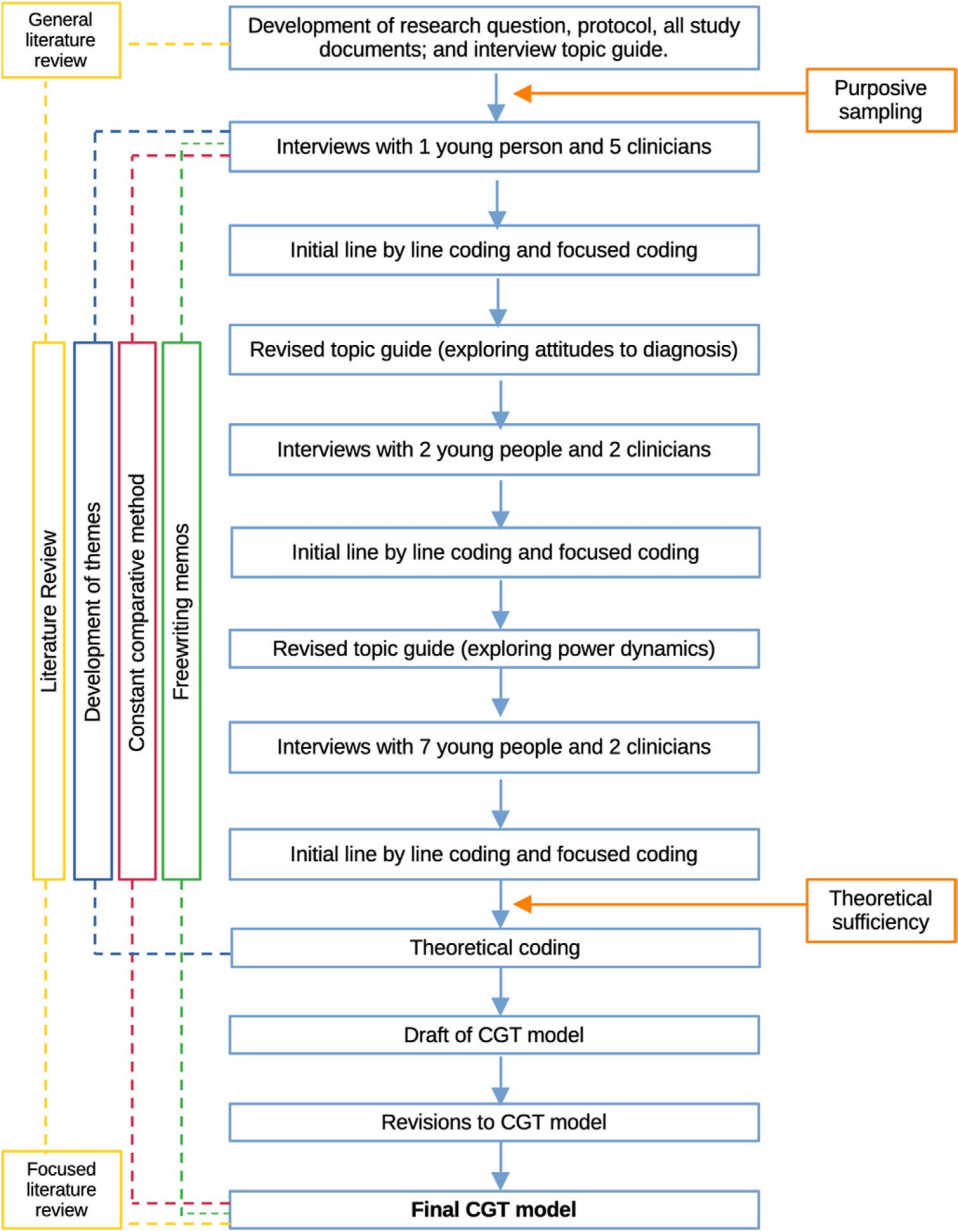
Flow diagram demonstrating CGT methodology.

Constructivist grounded theory (Charmaz, 2014, 2017, 2020) was used to analyse and synthesise the data. CGT is oriented towards patterns, meanings and processes within a specific context and is concerned with exploring relationships between relevant concepts. The process of construction is made transparent and it is recognised that findings are a construction from the researchers' understanding of the area being researched. CGT requires two stages of coding: initial coding and focused coding (Charmaz, 2014). Data were coded as they were collected. The primary researcher initially grouped meaningful units of data together at a descriptive level. Following this, the primary researcher entered into focused coding, which requires a more inferential approach. Across both styles of coding, the constant comparative method was used (Hallberg, 2006). Data were continually compared intraset and interset across the interviews. ‘Freewriting’ (Charmaz, 2014, p. 186), a strategy for writing memos with as little rules as possible was employed, in order to encourage intensive engagement and interaction with the data. The primary researcher immersed herself in purist CGT ideology in the writing of these memos, which insists that the researcher must ‘learn to tolerate ambiguity’ (Charmaz, 2014, p. 155). As the research question was concerned with formulation as a process between two parties, themes in the data which were captured across both datasets were of particular note. The primary researcher mapped themes from the young person data onto themes from the clinician data with respect to reciprocal moments in the formulation process. The process of coding and theory development was not linear, rather it moved back and forth from the raw data and reflective memos to the initial and focused codes. The second author was consulted throughout the focused coding and theory generation stage. As clinician/young person dyads were not recruited, clinician data were analysed with clinician data and young people data with young people data. A CGT approach allowed the researcher to read between the lines of the moment to moment processes to which participants were referring and map them onto each other across datasets. Charmaz's (2017) CGT coding process is said to be more ‘interpretive, intuitive, and impressionistic’ (Kenny & Fourie, 2015, p. 1279) than classic (Glaser, 1992) or Straussian GT (Strauss & Corbin, 1998) and this is seen in the coding and resultant theory of the present study. A pictorial representation of the CGT process is shown in Figure 1. Although grounded theorists typically aim for theoretical saturation, inductive thematic saturation, and a priori thematic saturation, it is often the case that researchers profess to have reached saturation, rather than presenting evidence for same (Charmaz, 2014; Leese et al., 2021; Saunders et al., 2018). It has been ventured that the concept of saturation is more appropriate for positivist leanings (Varpio et al., 2017). The creators of thematic analysis (Braun & Clarke, 2006) have declared the concept of data saturation neo‐positivist in orientation and not appropriate for a more constructivist orientation to qualitative research (Braun & Clarke, 2019). ‘Theoretical sufficiency’ may be a more appropriate term for a constructivist approach as employed here (Dey, 1999, p. 257), wherein the reflexivity of the researcher is acknowledged, and the notion that conjecture about data may never reach a point that is finite. A review of 100 grounded theory papers over a 7 year period showed the number of participants in the selected sample ranged from 5 to 114 (Thomson, 2010). The review tentatively purported that data saturation typically occurs after approximately 10 interviews. Rather than claim to meet data saturation and struggle to evidence this, the authors of the present study state that there was sufficient evidence in the data, codes, and categories, to propose a pilot CGT. A small sample is said to be justified if the findings of the analysis are rich in nature (Cleary et al., 2014) and the research team attest to the richness here. Relating to the write‐up, in the interest of transparency, the decision was made to present young people's and clinicians' experiences distinctly and discretely under each theme. Data were not mixed together in the write‐up so as not to obscure the positions of both cohorts. In doing this, key moments in the formulation process are interpreted, but the dyadic nature of the process upheld.

### Information about the research team

The primary researcher was a white female trainee clinical psychologist in her final year of her doctorate at the University of Limerick. During the write‐up stage of this paper, she was also completing a specialist placement at the host organisation. Alternatives to diagnosis and embodying a sense of shared humanity with clients are at the centre of her clinical work. The second author is a clinical psychologist who has worked as a clinical manager in the host organisation for the past 5 years. He approaches this work from a social constructionist perspective and is interested in non‐medical understandings of emotional wellbeing. The third author is a clinical psychologist and academic practitioner, with 27 years of practice experience who has previously published in the area of formulation as a framework for understanding psychological distress. The final author is a clinical academic professor and clinical psychologist with an interest in the process of formulation and its impact on outcomes.

## RESULTS

A CGT was developed with the data arising from the 19 interviews conducted.

### Participant characteristics

Nineteen participants were interviewed overall, 10 young people (male = 6, female = 4, age range = 16–23 years, mean age = 18.6, SD = 2.2), and nine clinicians from varying disciplines. These will be referred to individually as YP1 (young person 1), YP2 (young person 2), C1 (clinician 1), C2 (clinician 2) and so on. Details about the participants are given in the tables below (see Tables 1 and 2).

**TABLE 1:** Breakdown of participating clinicians.

	No. of years experience post‐qualification	Length of interview (min)
C1	7	50
C2	8	53
C3	5	61
C4	25	58
C5	2	45
C6	11	48
C7	2.5	38
C8	11	60
C9	2	56

**TABLE 2:** Breakdown of participating young people.

	Gender	Age (years)	Length of interview (min)
YP1	Male	20	31
YP2	Male	16	28
YP3	Male	20	53
YP4	Male	19	38
YP5	Male	16	39
YP6	Female	20	40
YP7	Female	16	23
YP8	Female	17	21
YP9	Male	23	56
YP10	Female	19	37

For the purpose of anonymity, the discipline of each clinician has not been stated in Table 1, but psychotherapy, occupational therapy, social work, mental health nursing, clinical psychology, counselling psychology, and educational psychology were all represented.

Although no one particular formulation approach was being studied, clinicians made reference to being trained in the following therapeutic orientations, theories, and frameworks: attachment based family therapy (ABFT), acceptance and commitment therapy (ACT), CBT, compassion focused therapy (CFT), dialectic behavioural therapy (DBT), emotion focused therapy (EFT), brief solution focused therapy, psychoanalytic therapy, narrative therapy, attachment theory, the interactive factors framework, the problem analysis framework, Rogerian principles, the multi‐perspective formulation model (5 Ps), the model of human occupations (MOHO), and the power threat meaning framework (PTMF). Uniting the practice of all clinicians was a focus on formulation without the need for a diagnosis.

### Overview of qualitative findings and model

**FIGURE 2:**
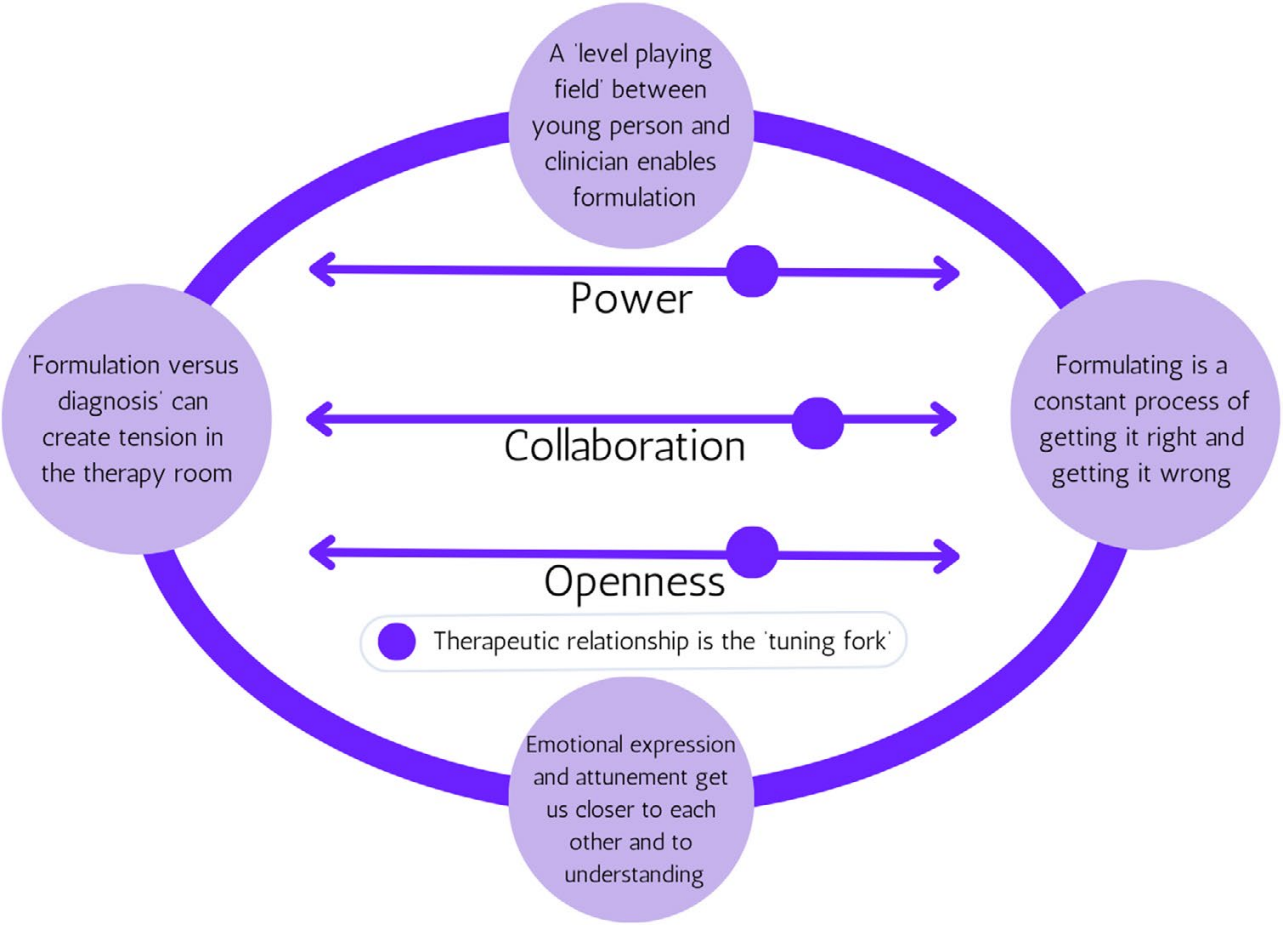
Constructivist grounded theory of dynamics underpinning formulation. The circles on the lines represent dials up and down on continuums. Picture the dial you would use to tune in an old radio. Their specific placement in this pictorial is not important. There is no inference regarding the direction or proportion of the constructs or their relationships. A circle unifies the themes on the outside and the continuums sit in the centre of the circle. This is to symbolise intersectionality. The themes on the circle are not sequential.

In analysing the data, some of the ideas in the literature (Halpin et al., 2016; Johnstone & Dallos, 2014; Thew & Krohnert, 2015) around formulation as an intervention became apparent. The generated theory conceptualises the dynamics likely to occur when young people and clinicians formulate together. The pictorial model below (see Figure 2) is not designed to tell the reader how formulation was experienced absolutely by participants, but to delineate some organising principles which can be used to reflect on the process. Dynamics are at the centre of the model, in that it emphasises that interactions can change from therapist to therapist, client to client, session to session, and moment to moment. The author proposes that formulation is a social process which takes place across the continuums of power, collaboration, and openness. These constructs are based on the research team's inductive interactions with the young person and clinician data. Placement on these continuums is impacted by the attunement of the therapeutic relationship. In this way, the therapeutic relationship acts as a ‘tuning fork’, taking stock of the dynamics between clinician and young person. Both the young person and the clinician rise and fall in terms of power, collaboration, and openness. These constructs are dynamic and can move from less to more and more to less in each moment of formulation as a process.

It is reasoned that the therapeutic relationship is the central tenet upon which all other concepts rely. The attunement of the therapeutic relationship determines whether the dynamics of power, collaboration, and openness are dialled up or dialled down. Four themes were generated from the data: A ‘level‐playing field’ between young person and clinician enables formulation, formulation is a constant process of getting it right and getting it wrong, emotional attunement gets us closer to each other and to understanding, and ‘formulation versus diagnosis’ can create tension in the therapy room. The therapeutic relationship, power, collaboration, and openness are represented in all themes. The model presented is intersectional in that it acts across each of the four themes. Young people's perspectives and clinicians' perspectives across each theme are presented.

#### Theme 1: A ‘level‐playing field’ between young person and clinician enabled formulation

The value of an even dispersal of power, and a sense of collaboration between young person and clinician is at the heart of this core category.

##### Young people's perspectives

Being on a ‘level‐playing field’ (YP10) with clinicians was important to young people,

It didn't feel condescending like ‘oh this is what you're feeling’ … It was more of a case she was just giving her opinion, and she was saying like ‘Tell me if I'm off the mark with anything’. (YP4)

The opportunity to correct their therapist and give their own opinion seems to have been empowering for young people.

I found, the stuff that he said, the way he brought it across, less of a ‘This is what you should do’, rather a ‘This is what I'd recommend’. (YP5)

This young person welcomed that their clinician was not overly prescriptive,

She gave me options to figure out myself, how I did it or why I did it… like it wasn't her pushing a reason almost. It was like giving me stepping stones to ‘why it is’, for me to figure it out myself… (YP6)

Being taught how to think rather than what to think seems to have been valuable for young people in collaborating on formulations ‘It was kind of like I was the artist or something and I was trying to paint a picture, and this was like the producer guy who was just kind of like looking at it and giving his thoughts on it as well’ (YP5).

All young people described having a collaborative relationship with their clinician, and it was noted that the work they did together with their therapist could not have been replicated alone, ‘We reached conclusions like together that I couldn't reach by myself at all’ (YP5).

Language was another feature which impacted power dynamics when formulating. Young people welcomed that their clinician did not use overly formal language, with one young person noting that her clinician did not use ‘clinical words’ or overly ascribe to ‘We're going to be doing CBT. You're going to do this, … and this’ (YP10).

Collaborating on formulations was made easier when young people did not perceive clinicians as leaning into the role of expert. The essence of this core category is captured in one young person's analogy, ‘It was like walking with someone as opposed to trying to catch up to them’ (C9).

##### Clinicians' perspectives

Not being seen to occupy an ‘expert role’ was important to clinicians when formulating. Clinicians made visible efforts to make young people feel empowered as the experts of their own lives,

I think you have to start from a position of curiosity and you have to start from a position of humility that we know what we know and we don't know what we don't. (C5)

It was also acknowledged that clinicians have training and experience in the field of mental health, but that the clinician and the young person need to team up for the work to be meaningful,

… we're both bringing our experience and our expertise here and we're going to kind of work on this together and make sense of things together. (C4)

It was important to clinicians that young people were fully involved in the process of formulating about themselves, ‘Nothing about you without you’ (C5).

Clinicians noted that it can be jarring when a young person or parent wants them to be ‘the expert’ and to hold their power in this way, ‘As much as I hate it, I do feel like they think you're meant to be the expert’ (C6). The expectation of expertise can put notable pressure on clinicians, ‘He was like, ‘Is this gonna work?’. And I'm like ‘I don't know’. And just being totally honest about that (C3).

All clinicians made some reference to how formulating in therapy seems to work better when it is guided by the young person, ‘I always feel it works better when I'm behind in it, if you know what I mean, when the person is leading it’ (C4). Although clinicians were mindful of not occupying an expert position, it was also acknowledged that as professionals, they had a duty to take an active role in working through formulations with young people,

It may not be enough to just follow the young person, but you must also show you are working hard with them… if we're truly collaborating with someone, we're not just a blank slate… We're bringing something … or offering a reflection. (C9)

Clinicians chose the formulation related language they used carefully, in an effort to best communicate with young people. Of the nine clinicians interviewed, six (C1, C2, C3, C4, C6, C8) noted that they had never used the word formulation with a young person. They did not want to alienate the young person with the use of clinical language, opting to be more ‘casual and sort of non‐threatening’ (C8). Clinicians found other ways to encapsulate the meaning of formulation using different terminology, ‘…I'd say … Let's try and find a way of making sense of this based on what you've gone through’ (C2).

Three clinicians (C5, C7, and C9) reported that they have, on occasion, used the word ‘formulation’ to describe their way of working to a young person. Age, capacity, and interest in psychology were named as determinants of whether or not a clinician would opt to use the word with young people. Using this terminology with young people is done in an effort to be wholly transparent about the therapeutic process, which can be said to minimise the power distance between young person and clinician.

#### Theme 2: Formulating is a constant process of getting it right and getting it wrong

The process of formulation requires some degree of agreement and disagreement until you find what is most ‘true’ for the young person. The terms ‘right’ and ‘wrong’ may appear to be positivist leaning, however they relate more to resonance than they do to accuracy. Essentially clinicians are trying to pinpoint what feels right and wrong for the young people.

##### Young people's perspectives

The integrity of this process was built on the young person's comfort with ‘correcting’ their clinician if they suggested something that did not fit. Being able to re‐direct their therapist if their formulation did not resonate with them was important to young people, ‘We developed that comfortable air. I could be like ‘Ehm I think you're a bit off now on that one’ (YP1). The ability to reject their clinician's suggestions seems to have been empowering for young people, ‘She made it very comfortable for me to say ‘no’ which was very … helpful’ (YP6). Equally, it can be frustrating for young people when clinicians put too much stock in their own formulation, without checking if it resonates with the young person,

And when I told (first male therapist) that, it kind of came up in every session… But I never thought it was as deep as bringing it up every single time. (YP9)

If the relationship between young person and clinician is not attuned, and the clinician keeps introducing formulations which do not reflect the young person's experience, a sense of exasperation with the process may creep in. Furthermore, the power distance between young person and clinician may become heightened, ‘It felt like he had almost all the answers and he was waiting for me to get to them’ (YP9). It seems that this process of playing ‘hot and cold’ was best received when young people's opinions were valued and they were given opportunities to reject hypotheses.

##### Clinicians' perspectives

All clinicians spoke of making gentle observations with young people to ‘see if it fits’, or ‘makes sense’. There seemed to be a sensitivity in the way clinicians did this, ‘It would be a very tentative, kind of suggestion, based on the relationship, and their readiness to hear it, and how it might impact them’ (C6). Checking in advance if a young person wanted to hear their clinician's view was a sensitive and subtle way to contribute to formulations,

I'll ask them if it's okay first or if they would like to hear … like a reflection on what they've been saying… (C9)

All clinicians also spoke of acknowledging to young people that their observations or suggestions may be wrong, and that they encourage young people to correct them if this is the case, ‘Just saying to them 'Will you let me know if I pick this up kind of wrong?'’ (C1).

Clinicians spoke of times when young people rejected their hypotheses and how they then took a new direction, ‘There'd be times when it's like, … no, that makes no sense to me’. I love it if they're able to challenge that … And if they say no, that's not it, we go away from that. I don't go back to that, y'know’ (C6). It seemed that clinicians liked being told ‘no’ as this was empowering for the young person, ‘That's efficacy in controlling your own narrative. I love to hear that’ (C5).

#### Theme 3: Emotional expression and attunement get us closer to each other and to understanding

It was shown that the process of formulation is more than trying to explain a young person's presentation. It is a dynamic relational process of expression and attunement. Moment to moment nuances and decisions led young people and clinicians closer together and closer to a sense of understanding.

##### Young people's perspectives

Clinicians' emotions were shown to have as much of a role as young people's emotions, and this was welcomed by young people, ‘We both expressed what we were feeling!’ (YP1). Expressing your emotions and having them validated was appreciated by young people, ‘[Clinician] allowed me to feel my emotions rather than trying to hyper‐analyse them by ‘literally sitting in the emotions’ (YP10). In a well attuned relationship, young people felt that their clinicians could detect what they ‘needed to’ talk about, even if they were avoiding it slightly,

She would sway the conversation one way … and like ask you particular questions that could be … like sensitive towards me and I'd get like a bit upset, … I feel like she … knew what I wanted to say, but that maybe I wasn't saying it, so she'd … steer the conversation that way to kind of ask me about the main problem (YP8)

Overall, formulation appeared to be a cathartic process for most young people. How young people's emotions were handled informed the development of the relationship and the development of the formulation.

##### Clinicians' perspectives

Clinicians strived to set up a space where young people could express emotions safely, and described using different therapeutic approaches with different young people. Clinicians are constantly making moment to moment decisions on how best to respond to young people, ‘I'm just kind of a tuning fork’ (C5).

Connecting with the young person was paramount in creating a space where formulation could happen,

It felt like a very good connection. It felt quite profound really to be honest. But in that moment, you nearly forget that you're a clinician, you know. For me, it was just two human beings really sharing something and connecting on a really deep level. (C4)

Clinicians used their intuition to gauge young people's reactions to formulations, ‘There was just kind of a lightbulb for me and it just felt like it really resonated with the young person’ (C8). A young person's readiness to formulate and the timing were always being considered by clinicians, ‘… the image in my head was just this sense that you could unravel if you take it all out too much. You need to be at a place to be able to do that’ (C4).

#### Theme 4: Formulation versus diagnosis can create tension in the therapy room

The interviews brought up ideological issues around diagnosis and formulation. These opposing viewpoints sometimes resulted in an ‘edginess’ in the work.

##### Young people's perspectives

Young people differed in their opinions on diagnosis and formulation. Some young people reported that they did not believe in diagnoses or ‘a label’ (YP1). Some young people reported going on a journey of understanding, with some even reporting that they felt like a ‘new person’ (YP9) following therapy,

I was like very very different. I feel like I understand why I have certain feelings or I feel certain things much more. (YP9)

Despite this, this young person still wanted a diagnosis following therapy, as they felt it might provide some validation,

To be honest, I wanted it… I think it's because I was really like exhausted from feeling like a certain type of way for like years and years and not having … that like label… I just wanted someone to validate what I was feeling. (YP9)

##### Clinicians' perspectives

Clinicians made reference to the fact that they opted to work in the host organisation as it does not position itself within a medical model, and this permits them to practice in a way that was in line with their values. Based on clinicians' reports, formulation seemed to be suitable when a young person had a curiosity about the source of their difficulties. Clinicians noted that they might give the young person the opportunity to explore their difficulties via formulation, but that if they were not interested, they would make a referral to the appropriate service,

They might say… ‘I need a diagnosis’ and … we don't do diagnosis, so this is our understanding and it may not fit for you but you can see what you make of it. And if you really feel you need that, like you need to kind of look at that diagnosis … we can link you in with your GP. (C2)

It was difficult for clinicians, who have reservations around the concept of psychiatric diagnosis, to work with clients in a theoretical framework that was inconsistent with their beliefs and training,

There have been some people that have come and said ‘I want you to tell me I have clinical depression’ … and that is hard because there's a reason they want that, so I have to respect that. (C6)

One clinician recalled a case where a young person had sought a diagnosis and he had discouraged her from pursuing this and tried to encourage the development of a formulation instead. This resulted in the young person disengaging with the service,

I told her ‘I think I owe you an apology. I wasn't listening to you and what you were talking about. Would you come back for a final session and maybe we can talk about how we can help you and get what you're looking for?’ So she did come in and I sent a referral letter to the GP for assessment with CAMHS … It broke my heart. (C3)

The opposing principles of diagnosis and formulation may cause some rifts in practice. These may be largely unspoken.

## DISCUSSION

The present study proposes a grounded theory continuum model wherein young people's and clinicians' power, collaboration, and openness are constantly in flux over the course of the process of formulation. It is proposed that the therapeutic relationship, however attuned it is, impacts each of these constructs. The theory above presents a dial model, where power, collaboration, and openness are dialled up and dialled down, often subconsciously from moment to moment when formulating. It has been suggested that the therapeutic relationship may act as a vehicle for change for clients (Vilkin et al., 2022). Herein it is suggested that the attunement of the therapeutic relationship informs the clinician what is needed by the client. Similarly, throughout the process of formulation, clinicians are making moment‐to‐moment decisions in responding to young people's needs, and this is represented in the continuum model presented.

The present study conceptualises formulation as a process of ‘getting it right and getting it wrong’. The entire process of formulation is built on creating a relationship secure enough that disagreement or ‘resistance’ does not rattle the entire relationship. It is a welcome and encouraged facet of understanding and taking control of one's own narrative. In writing disagreement into the process, opposing opinions become a normal part of the social process of back and forth, and not an aberration.

Halpin et al.'s (2016) study on young people's experiences of formulation reported that young people found the process of formulation emotionally evocative, a finding which was mirrored in the present study. In reflecting on past emotions, you conjure up live emotions, which can then be acted on and interpreted in the therapy space. This finding may encourage clinicians not to be limited to consolidating one ‘true’ or ‘accurate’ formulation, as there is constantly new data emerging to test the formulation. Although Halpin et al. (2016) also focused on young people's and clinicians' experiences of formulation, a novel aspect of the present paper is its discourse around power and openness, which the findings show to be paramount when working with younger clients.

The findings of the present study relate to but also build on two previous grounded theory studies (Gibbs et al., 2020; Rayner et al., 2011) in the area of formulation. Rayner et al. (2011) note that cognitive analytic reformulation was seen as important by clients but only when ‘embedded’ in the context of a ‘trusting and collaborative therapeutic relationship’ (p. 299). The present study also found formulation to be embedded into the therapeutic alliance. Rayner et al.'s (2011) model entitled ‘Doing with’, mirrors the sentiment of collaboration shown in the core conceptual framework of the present study. Their core conceptual framework acknowledges formulation as a vehicle for feeling, not just understanding. The present study also emphasises the emotions explored but also arising from formulation. The importance not only of understanding, but of relating and feeling was also captured in Gibbs et al.'s (2020) grounded theory on formulation. Despite the similarities between the present grounded theory and the two that have gone before, there were also some notable differences. The present study was constructivist in epistemology, by comparison to Gibbs et al. (2020) who adopted a critical realist viewpoint, and Rayner et al. (2011) who did not disclose their stance. A constructivist position allowed the present team more abstraction than that afforded by other ontological positions. Consequently, the analysis read between the lines of the data to speak the unspoken, ie: young people and clinicians did not explicitly extrapolate relationships between power, collaboration, and openness, but provided the data which allowed the research team to be active agents in their interpretation of the data. As clinicians utilise the language of formulation but young people do not, this extra layer of inference and advocacy was necessary to truly allow young people's voices to contribute to the conversation on formulation, a conversation that is often reserved for clinicians. Also, the present study reported findings on the attrition between formulation and diagnosis, a discourse not acknowledged in previous grounded theory studies in the area.

Formulation is consistently recommended to be a collaborative process (British Psychological Society, 2011; Johnstone & Dallos, 2014). The findings of the present study do much to capture the value of true collaboration as opposed to didactic prescription. It has been suggested that young people may view clinicians as authority figures which naturally has implications for the power dynamics in the therapy room (Pearce & Sewell, 2014). The power of the clinician in CBT, in particular, and their use of this power has been questioned (Proctor, 2008), as the core principles of CBT position the clinician as the expert who ‘corrects’ the clients ‘thinking errors’. If loss of personal power is a major contributor towards psychological distress (Proctor, 2002), the possibility that therapy could further serve as a power vacuum for clients is concerning. The present model highlights the value of formulating ‘with’ as opposed to formulating ‘at’. The findings of the present study, most notably the themes ‘A level playing field between young person and clinician enables formulation’ and ‘Formulation is a constant process of getting it right and getting it wrong’ showcase how young people appreciate an even dispersal of power in the therapeutic relationship. In choosing which therapeutic modalities to apply to cases, the findings of this study suggest that therapists must interrogate power dynamics which may be inherent in particular approaches, and consider how to tailor these with young people in mind.

Results relating to difficulties arising from a clash between conceptualisations of mental health (diagnosis versus formulation) were mirrored in Hood et al.'s (2013) thematic analysis of clinicians' experiences of formulation. Participants in this study expressed anger at the prevalence of the biomedical model, and a belief that it was inherently harmful. While clinicians and some young people in the present study mirrored these ideas, it was important to note that some young people reported finding meaning in biomedical models. Differences in opinion between client and clinician are bound to happen with regard to meaningful formulation but it is how these are handled that determines whether or not the client finds the exchange therapeutic. Collaboration and openness is needed on both sides to mitigate the effects of opposing viewpoints.

### Methodological strengths and weaknesses

As highlighted previously, formulation is poorly defined (Christofides et al., 2012; Thrower et al., 2024). This is especially the case for clients as this study highlights that clinicians may not typically use this term with young people. However, the young people who participated were aware of the nature of the research on formulation and self‐selected in to the study. In addition, as clinician/young person dyads were not recruited, the analysis between clinician and young people's views was more of an abstraction, as opposed to the experiences of one therapeutic pairing. Imposing the need for therapeutic pairings may have set up a dynamic of obligation around participation, and it was essential that candidates participated of their own free will. Some of the clinicians interviewed had worked with some of the young people interviewed, and some had not. However, interviewing clinicians and young people who had not necessarily worked together is not thought to have impacted on the research team's ability to answer the research question. Furthermore, this approach allowed the team to enquire as to clinicians' broad experience, as opposed to the exchanges in one therapeutic pairing. This engendered a richness of experience in the data. Information re ethnicity, socioeconomic status, sexual orientation, and gender identity were not sought for this study. This should be taken into account when considering the findings as marginalisation undoubtedly has an impact on power dynamics. Resources did not allow for transcripts to be inter‐rated for accuracy and this could be seen as a limitation. As formulation as a unifying core competency across numerous therapeutic ideologies was explored, it may be seen as a shortcoming that more specification was not required regarding specific therapeutic modalities. However, this can also be viewed as a strength, as the eclectic and integrative nature of ‘treatment as usual’ is acknowledged. The fact that participants came from routine clinical services may increase confidence in the verisimilitude and ecological validity of the findings, and this can be considered a strength. Clinicians interviewed came from a transdisciplinary team, and clients were transitional aged youth, both of which tend to be significantly under‐represented in the research on formulation.

### Clinical implications

The findings of the present study have implications for how clinicians formulate with young people in mental health services. One finding which may have implications for service delivery is young people's perception of ‘expertise’ or formality as a barrier to connection while formulating. This may be particularly pertinent in more traditional clinical service settings. Furthermore, the finding that formulation is a process of trial‐and‐error shows the problem of imposing time constraints on formulation: such time pressures are not uncommon in triage/screening sessions.

There is potential for the continuum model presented herein to have clinical utility in youth mental health services which utilise formulation. While ultimately we work to empower and collaborate with our clients, this study has shown that sometimes young people want the therapist to hold their power as a clinician, or may want their therapist to lead sessions. This can be challenging for clinicians. It is hoped that the CGT presented here acts as a reflective practice tool that can be used by clinicians and supervisors in considering these dynamics when they arise.

The theme ‘Formulation versus diagnosis' can create tension in the therapy room’ highlights the ideological barriers that may present between young people and mental health services. The findings of a systematic review on how psychiatric diagnosis affects young people's self‐concept and social identity reported that diagnosis may threaten this cohort's self‐concept, and lead to social alienation (O'Connor et al., 2018). However the same study reported that young people may also find self‐understanding and acceptance in a diagnosis. Conversely, adolescents diagnosed with depression have reportedly struggled with the notion that experiencing negative emotions constitutes a ‘disorder’ and have interpreted it as a ‘reification’ of abnormality (Viduani et al., 2022). Clearly there is tension in the literature around the suitability of diagnosis for this population, and this same tension presents in the therapy room. Rather than weigh in heavily on this argument, as would be beyond the scope of this paper, this paper presents a theoretical model which may prove useful when this tension in ideology presents in the therapeutic space, ie: reflection around power imbalances, collaboration, the young person's openness, and the clinician's own openness is in and of itself a tension reducing exercise.

### Future research

The present study considered clinicians and young person's views of the process of formulation, however when working with young people, one is often working with the family too. A qualitative study of parents' perspectives of the formulation process for their young people would shed light on how the greater system interacts with devising and putting a formulation to use. There is also a dearth of pre and post study designs which explore clients' personal understanding of their difficulties prior to formulation, and their perspectives post formulation. As the present study explored transitional aged youth's experiences of formulation, there is scope to explore the experiences of younger adolescents in future studies. The present study was conducted in one youth mental health organisation nationally, further studies in different organisations in different countries would shed light on the generalisability of results. While CBT and CAT are represented in previous studies on formulation, the present study viewed formulation as a core competency across numerous therapeutic modalities and so did not specify which model of formulation was used. In light of this, a natural next step is to consider how transitional aged youth experience formulation in specific modalities. Lastly, this study did not enquire as to participants' socio‐economic status, sexual orientation, or ethnicity. As the findings of this study indicate that power, collaboration, and openness fluctuate during the process of formulation, further study is needed on how marginalised populations experience the process, as they may be more likely to experience inequity.

## CONCLUSION

This study suggests that formulation is a social process with variety, relational elements, challenging emotions, trial and error, and shared humanity. Analysis of the voices of transitional aged youth and clinicians in this study have shown that formulation comprises dynamics of power, collaboration, and openness, held within an attuned therapeutic relationship. The constructivist grounded theory analysis has generated key organising principles that will hopefully have additional clinical utility for clinicians and supervisors in considering the social phenomena that occur during formulation with young people.

## AUTHOR CONTRIBUTIONS

**Laura J. Douglas:** Conceptualization; formal analysis; visualization; writing – original draft; methodology; investigation; project administration; writing – review and editing; software. **Cian Aherne:** Conceptualization; formal analysis; methodology; supervision; writing – review and editing; writing – original draft. **Patrick Ryan:** Supervision. **Barry Coughlan:** Conceptualization; supervision. **Donal G. Fortune:** Conceptualization; formal analysis; writing – original draft; methodology; supervision; project administration; writing – review and editing.

## CONFLICT OF INTEREST STATEMENT

The authors have no conflict of interest to declare.

## Data Availability

The data that support the findings are not publicly available due to ethical restrictions.
